# Antioxidant Properties and Microbiological Stability of Yogurt Enriched with Elderberry Extract

**DOI:** 10.3390/foods14071251

**Published:** 2025-04-03

**Authors:** Oana-Elena Pascariu, Letícia M. Estevinho, Natália L. Seixas, Ioan Dopcea, Oana A. Boiu-Sicuia, Mihaela Geicu-Cristea, Florentina Israel-Roming

**Affiliations:** 1 Faculty of Biotechnologies, University of Agronomic Sciences and Veterinary Medicine of Bucharest, 59 Mărăști Blvd, District 1, 011464 Bucharest, Romania; oana.sicuia@bth.usamv.ro (O.A.B.-S.); mihaela.geicu@usamv.ro (M.G.-C.); florentina.israel@bth.usamv.ro (F.I.-R.); 2 Centro de Investigação de Montanha, Instituto Politécnico de Bragança, 5300-253 Bragança, Portugal; leticia@ipb.pt (L.M.E.); natalia.seixas@ipb.pt (N.L.S.); 3 CEBIS International, 47 Bd. Theodor Pallady, 032275 Bucharest, Romania; ioan.dopcea@gmail.com

**Keywords:** *Sambucus nigra*, elderberries extract, bioactive compounds, yogurt, shelf life

## Abstract

This study examines the impact of added elderberry (EDB) extract on the physico-chemical, microbiological, and sensory properties of yogurt over a 21-day storage period. Two separate experiments were conducted: the first focused on testing concentrations of 0.05%, 0.1%, 0.2%, and 0.5% (*w*/*w*) EDB extract for impoving antioxidant properties and replacing potassium sorbate, a chemical preservative commonly used in dairy products, while the second aimed to enhance the bioactive compounds’ concentration by adding 0.5% EDB extract, and to assess the effect of a sweetener (agave syrup) on the sensory profile. Both experimental approaches demonstrated a significant increase (*p* < 0.01) in total phenolic content. In the first experiment, there was approximately 2.6 times more total phenolic content compared to the control (with a maximum of 11.71 mg GAE/100 g for the sample with 0.2% EDB extract), and about 6 times more with the addition of 0.5% EDB extract and agave syrup (with a maximum of 25.29 mg GAE/100 g). Additionally, the IC_50_ value decreased for all samples with EDB extract, suggesting enhanced antioxidant activity. Specifically, the antioxidant activity was approximately 3.3 times higher for the control for samples obtained from homemade yogurt with EDB extract, and about 8 times higher for samples obtained by adding EDB extract to commercial yogurt, compared to their respective controls. The high stability of bioactive compounds during the storage period highlights the potential of EDB extract as a functional antioxidant ingredient. Microbiological analysis confirmed the safety of the yogurt, with lactic acid and mesophilic bacteria showing stable counts and minimal declines over time. In the first experiment, a reduction of about 2.3 CFU/g was observed from day 0 to day 21 in samples with 0.1% and 0.2% EDB extract, while the control sample showed a reduction of 1.84 CFU/g. However, the control sample recorded the growth of psychrophilic bacteria, yeasts, and molds. In the second experiment, the samples with 0.5% EDB extract showed an average reduction of approximately 0.35 CFU/g, while the control showed a reduction of 0.51 CFU/g, maintaining stable counts and no detectable growth of psychrophilic bacteria, yeasts, molds, or coliforms. Additionally, the inclusion of different concentrations of EDB extract, along with the combination of a higher concentration of EDB extract and agave syrup, improved the yogurt’s sensory attributes, thereby enhancing consumer acceptance. For the first experiment, 68% of the panelists expressed their preference for the samples with EDB extract, and 10% preferred the control sample. In the second experiment, 49% preferred the sample with 0.5% EDB extract and agave syrup, while 23% preferred the control sample. These findings support the integration of EDB extract into yogurt formulations to boost antioxidant properties while preserving microbiological stability. Future research should explore the potential health benefits and long-term effects of these functional dairy products.

## 1. Introduction

*Sambucus nigra* L. (*Adoxaceae* family), sometimes referred to as black or European elderberry, is a shrub that extensively grow over Europe, West and Central Asia, and North Africa [1]. The utilization of elderberries (EDBs) has gained significant attention in recent decades, attributed to their distinctive flavor, complex chemical profile, widespread occurrence in natural ecosystems, and their suitability for cultivation [2]. The vibrant purple and red coloration of EDBs is attributed to the presence of anthocyanins, a group of natural pigments. These pigments have contributed to the use of EDBs as natural dyes in various industrial sectors [3]. Polyphenols represent the main bioactive compounds with a potential beneficial effect on the human body [4,5,6]. The polyphenolic content in regularly consumed fruits and vegetables varies depending on factors such as species, cultivation conditions, and ripeness. The development of functional foods fortified with a high concentration of antioxidants is essential, as conventional diets often do not meet the required antioxidant intake [7]. To enhance health and quality of life, it is essential to develop a wide range of functional foods—natural, antioxidant-rich products with potential chemopreventive, immunostimulatory, atheroprotective, and anti-inflammatory properties. Current trends in the food industry are orientated towards reformulating functional products to meet consumer expectations, namely, to improve traditional products and replace synthetic additives. According to this perspective, the abundance of bioactive compounds, antioxidants (phenolic compounds), organic acids, and vitamins make EDBs a highly valuable material for biotechnology, food technology, and pharmaceutical industry [8]. EDBs are generally underutilized and rarely incorporated as food ingredients. An explanation could be given in the fact that harvesting the fruits is a difficult process. Harvesting EDB is difficult due to several factors. The plants are tall and hard to reach, so the fruits must be picked manually. Also, the berries ripen in a short period, which makes timing very important. If not handled properly, the fruits can be damaged, leading to a loss of quality. Food processing includes a series of steps and parameters that could affect the stability of anthocyanins. These compounds have low stability under certain circumstances, including exposure to light, oxygen, heat treatments, pH variations, etc. [9,10]. However, numerous studies have demonstrated the positive health effects of EDBs. The most important of these effects are the antioxidant activity [11,12], as well as antidiabetic and anticancer effects [13,14,15], and antibacterial and antiviral properties [16,17,18,19].

The use of EDBs has been studied in various food products, such as juice, tea, liquor, and spread [20], wine [21], jam, crumble, muffins, mousses [22], fiber-enriched pasta [23], croissants [24], gluten-free wafers [25], meat [26,27], and dairy products [28,29,30]. The temperature at which the products are prepared, the time of exposure to high temperature, the pH and the chemical composition of the product are important factors in the preservation of bioactive compounds, which could have therapeutic potential. Considering the low pH of fermented dairy products and the stability of anthocyanins at acidic pH, along with the fact that the addition of fruit extract can be made after the heat treatment of raw milk and after the fermentation process, the development of dairy products with EDB extract may be considered as a way to introduce EDBs into the daily diet. Conventional yogurt, which is defined as milk that has been fermented using bacterial strains, is a source of probiotics that have been shown to improve in vivo lactose digestion. Yogurt is a valuable food for individuals who may lack certain nutrients, such as children and the elderly. The fermentation process of milk can boost the bioavailability of nutrients, such as vitamin B-12, calcium, and magnesium, among others, as well as proteins and peptides [31,32]. Foods have a complex composition and are usually consumed in combination with other foods, making it difficult to determine the specific health benefits of each individual food. This is further complicated by the fact that certain compounds may interact through positive or negative synergistic effects. In recent years, consumer preferences have increasingly shifted toward clean-label and minimally processed foods, with a growing interest in natural alternatives to synthetic additives [33]. Within the dairy sector, this trend has driven research and innovation aimed at identifying natural preservatives that not only ensure microbial safety, but also align with consumers’ demands for healthier and more sustainable products. Plant-based extracts, rich in bioactive compounds such as polyphenols and flavonoids, are gaining popularity due to their multifunctional properties, including antioxidant and antimicrobial activity. Combining fruits and dairy products in one product can provide the daily intake of nutrients for people who consume these food categories in suboptimal proportions. Both EDBs and yogurt contain a wide variety of bioactive compounds. Their proportion can decrease or increase depending on several factors such as the period and place of fruit harvesting, the degree of ripening, processing methods, period and storage conditions, as well as milk quality, fermentation method, and the bacterial strain used. These aspects make it very difficult to assess the positive effects on human health when comparing one yogurt type to all yogurts in the same category [34]. Given these complexities, it is essential to consider nutritional, safety (microbiological and toxicological), sensory, and commercial (price) aspects when producing dairy foods containing herbal extracts [35].

This study aimed to develop a new yogurt variety enriched with bioactive compounds derived from EDB extract while preserving the viability of beneficial microorganisms. The study was conducted in two phases, each with a distinct objective.

In the first phase, we focused on investigating the possibility of replacing a chemical preservative (potassium sorbate) with a natural alternative (EDB extract). Potassium sorbate is commonly used to extend the shelf life of yogurt by inhibiting the growth of yeasts, molds, and other spoilage microorganisms. Various concentrations of EDB extract were tested, and physico-chemical analyses were carried out to assess whether small amounts of the extract could introduce beneficial compounds, such as antioxidants, without negatively affecting the viability of beneficial microorganisms and sensorial properties.

In the second phase, we aimed to enrich the yogurt with a higher concentration of bioactive compounds by increasing the amount of EDB extract, and tested the effect of adding agave syrup as a sweetener. The addition of agave syrup was considered to assess its impact on the sensory profile and to explore its potential prebiotic effects in combination with the EDB extract.

These two phases of the study were designed to address different research questions and focus on distinct aspects of yogurt formulation, preservation, and enrichment. Therefore, they should be considered separately, as the objectives and experimental setups differed from one phase to another.

## 2. Materials and Methods

### 2.1. Chemical and Reagents

The following media and reagents were used: Dicloran Rose Bengal Chloramphenicol (DRBC) Agar (Carl Roth GmbH + Co. KG, 89, Karlsruhe, Germany), Man, Rogosa and Sharpe (MRS) Agar (HiMedia, Mumbai, India) and PCA Biocontrol™ (Bellevue, WA, USA). Analytical-grade substances included potassium sorbate (C_6_H_7_KO_2_; Sigma-Aldrich, St. Louis, MO, USA), sodium carbonate (Na_2_CO_3_; Sigma-Aldrich, St. Louis, MO, USA), sodium acetate (CH_3_COONa; Merck KGaA, Darmstadt, Germany), and aluminum chloride (AlCl_3_; Thermo Fisher Scientific, Waltham, MA, USA). The standards used were Folin–Ciocalteu reagent (Merck KGaA, Darmstadt, Germany), 2,2-Diphenyl-1-picrylhydrazyl (DPPH) reagent (Sigma-Aldrich, St. Louis, MO, USA, Germany), rutin (Carl Roth GmbH + Co. KG; Karlsruhe, Germany), gallic acid (Sigma-Aldrich, St. Louis, MO, USA, Germany), and ascorbic acid (Merck, Darmstadt, Germany, China). The extraction solvent used was ethanol (Sigma-Aldrich, St. Louis, MO, USA).

### 2.2. EDB Extract Procedure

The elderberries of *Sambucus nigra* subsp. *nigra* used for the extract were harvested at full ripeness from the wild flora of the northeastern part of Romania (46°51′55″ N 26°54′48″ E), where the soil is of the chernozem type. After harvesting, the berries were separated from their pedicels, as well as from unripe, overripe, and dry berries, and stored in plastic bags before being frozen at −18 °C. The frozen berries were lyophilized for 48 h at −50 °C and 0.01 mbar, resulting in a water content of 5.4%, using a laboratory freeze-dryer (Labconco 6, Labconco Corporation, Prospect Ave, Kansas City, MO, USA). The lyophilized berries were ground and stored in dark glass containers at room temperature until further use.

The extract was obtained using optimal working parameters, as outlined in the method described by Pascariu et al. [36], which involved ultrasonic extraction for 40 min with a 45% ethanol solution, at a 1:100 (*w*/*v*) ratio. After extraction, the liquid was concentrated using a rotary evaporator (R-215 Rotavapor, Vacuum Controller V-850; Büchi Labortechnik AG, Flawil, Switzerland) and lyophilized using the same laboratory freeze-dryer (48 h at −50 °C and 0.01 mbar), with the final product having 83.35% dry matter being stored in the refrigerator (4 ± 2 °C).

### 2.3. Yogurt Production

**Figure 1:**
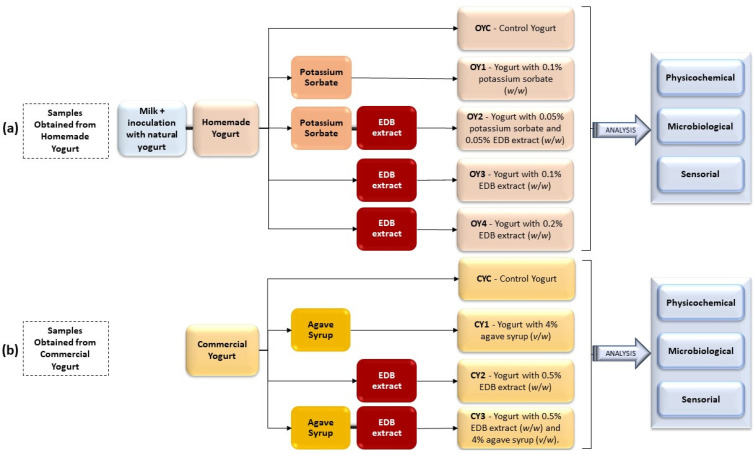
Schematic representation of yogurt preparation for two experiments: (**a**) Samples obtained from homemade yogurt; (**b**) samples obtained from commercial yogurt. The figure also shows the categories of analyses performed on each sample.

The use of EDB extract as an ingredient in yogurt preparation aimed to improve the product’s nutritional profile and to replace a chemical preservative (potassium sorbate) with a natural extract that inhibits the growth of undesirable microorganisms. This may enable the preserving of the probiotic properties of the yogurt (i.e., maintaining the beneficial microflora) while achieving a high level of consumer acceptability. To accomplish these objectives, two approaches were applied, corresponding to two different yogurt bases used in the study: one prepared in the laboratory and the other commercial (Figure 1).

#### 2.3.1. Samples Obtained from Homemade Yogurt

This approach involved using milk as the raw material through to the finished yogurts, incorporating either a chemical additive or EDB extract. This experiment was carried out in the Microbiology laboratory of Instituto Politécnico de Bragança, using milk, powdered milk, and commercial natural yogurt as inoculum, all sourced from Portugal. For yogurt production, semi-skimmed ultra-high-temperature (UHT) processed milk (Continente) was used. According to the nutritional information provided on the product label, the average composition per 100 mL was as follows: 1.6 g fat (of which 1.0 g saturated fat), 4.9 g carbohydrates (of which 4.9 g sugars), 3.4 g protein, and 0.1 g salt. In Portugal, UHT milk is the most readily available, and has been utilized in several earlier studies on yogurt production [37,38]. The inoculum consisted of natural yogurt labeled as “containing live and active cultures”, with a concentration of approximately 10^8^ colony-forming units (CFU) per gram. According to the manufacturer, the starter cultures in the yogurt used as inoculum included *Lactobacillus bulgaricus*, *Streptococcus thermophilus*, and *Bifidobacterium lactis*. The yogurt used was a commercial product (Danone Activia), with the following average composition per 100 g: 3.8 g fat (of which 2.3 g saturated fat), 3.9 g carbohydrates (of which 3.9 g sugars), 3.3 g protein, and 0.1 g salt. The yogurt-making process was conducted under sterile conditions. One liter of UHT milk was heated to 50 °C and mixed with powdered milk (Molico from Portugal, 1.0%, *w*/*v*; composition per 100 g: 0.7 g fat, 50 g carbohydrates, 34.5 g protein, 1.1 g salt) to ensure optimal dissolution. The mixture was then cooled to 42 ± 2 °C and inoculated with yogurt (12%, *w*/*v*). The sample was incubated in a thermostatic bath for 8 h at 42 ± 2 °C to allow the development of bacterial cultures [39]. The pH of the yogurt was 4.10 immediately after fermentation. The yogurt was placed in the refrigerator (4 ± 2 °C) for 48 h to slow down bacterial activity and maintain product stability. The obtained yogurt was homogenized and divided into five samples. The EDB extract was prepared as described in Section 2.2, redissolved, and added to the samples OY2, OY3, and OY4. The composition of the extract was analyzed to determine the concentrations of total polyphenols, total flavonoids, and other bioactive compounds. The results of these analyses are presented in Section 3.1. Five samples were obtained, as shown in Figure 1, as follows: control yogurt (OYC); yogurt OY1 with potassium sorbate (0.1%, *w*/*w*); yogurt OY2 with 0.05% potassium sorbate and 0.05% EDB extract (*w*/*w*); yogurt OY3 with EDB extract (0.1%, *w*/*w*); yogurt OY4 with EDB extract (0.2%, *w*/*w*). Each sample was divided into four portions for physicochemical and microbiological analyses at four different time intervals: 0, 7, 14, and 21 days. The samples were stored in a refrigerator (4 ± 2 °C) for further analysis.

Although the protocol was described per one liter of milk for clarity and reproducibility, all experiments were carried out in triplicate. Additionally, for the sensory evaluation, a separate batch of approximately 8 kg of yogurt was prepared, following the same formulation protocol, and divided into five samples (OYC, OY1, OY2, OY3, and OY4), to ensure sufficient quantities for organoleptic assessment.

#### 2.3.2. Samples Obtained from Commercial Yogurt

This approach involved using commercially available yogurt with live lactic cultures (*L. bulgaricus*, *S. thermophilus*, and *B. animalis* subsp. *lactis*), labeled as “containing live and active cultures” (3.2 × 10^9^ CFU/100 g), to produce yogurts with EDB extract and agave syrup. The experiment was conducted in the Microbiology laboratory of the Faculty of Biotechnology, University of Agronomic Sciences and Veterinary Medicine of Bucharest, using materials from Romania. In this experiment, the use of commercial yogurt as a standardized base ensured product consistency and facilitated the assessment of the impact of increasing the percentage of EDB extract in a product with a 21-day shelf life. The yogurt used as the base for the formulation was a commercially available strained yogurt (Danone Activia), with the following average composition per 100 g: 3.4 g fat (of which 2.2 g saturated fat), 3.9 g carbohydrates (of which 3.9 g sugars), 3.5 g protein, and 0.12 g salt. To evaluate the extract’s maximum potential and improve the chemical profile of the yogurt, the concentration of EDB extract was increased to 0.5% (*w*/*w*). This adjustment was made to assess the effects of a higher extract dose on the functional and organoleptic properties of the product. The same EDB extract as described in Section 2.3.1 was used in the second experiment.

Agave syrup, a natural sweetener, can positively influence the product’s market acceptability by enhancing the yogurt’s palatability and aligning with current consumer trends toward healthier foods. The agave syrup used in this study was a commercially available product (Nature’s Promise). According to the nutritional label, the average composition per 100 mL was as follows: 106 g carbohydrates (all of which were sugars, primarily fructose), and 0 g fat, protein, and salt.

To investigate the individual and combined effects of EDB extract and agave syrup, the production process involved homogenizing the yogurt and then supplementing it—depending on the experimental formulation—with EDB extract (0.5%, *w*/*w*) and/or agave syrup (4%, *v*/*w*). Four yogurt samples were prepared, as shown in Figure 1: a control yogurt (CYC); yogurt with agave syrup (CY1; 4%, *v*/*w*); yogurt with EDB extract (CY2; 0.5%, *w*/*w*); and yogurt with both EDB extract and agave syrup (CY3; 0.5%, *w*/*w*; 4%, *v*/*w*). Each sample was divided into four portions for physicochemical and microbiological analyses at time intervals of 0, 7, 14, and 21 days, and stored in a refrigerator (4 ± 2 °C) until further analysis.

All formulations were prepared in triplicate to ensure reproducibility and statistical analysis. Additionally, 8 kg of commercial yogurt were prepared for sensory evaluation, equally distributed among the four samples (CYC, CY1, CY2, and CY3), providing sufficient quantity for sensory testing.

### 2.4. Physicochemical Analysis

#### 2.4.1. pH Value

The yogurt samples were homogenized, and their pH was measured at intervals of 0, 7, 14 and 21 days after production. pH measurements were performed at room temperature using a potentiometer with a combined pH electrode (Mettler Toledo, Mumbai, India). All measurements were performed in triplicate, and the results were expressed as the mean value ± standard deviation (SD).

#### 2.4.2. Color

The color characteristics of the yogurts were determined immediately after production, using a colorimeter (CR-400 Chroma Meter, Konica Minolta Inc., Osaka, Japan), according to the CIE L*a*b* color metrics. Before each measurement, the device was calibrated using a white calibration plate to ensure the accuracy of the results. The yogurt samples were placed in a clear container and homogenized to eliminate any texture variations that could affect the color reading. The L* value represents lightness (0 = black, 100 = white); the a* value ranges from −a (greenness) to +a (redness); the b* value ranges from −b (blueness) to +b (yellowness). Chroma (C*) refers to the intensity or saturation of color, representing how vivid or pure the color appears, and can be calculated using Equation (1). A higher chroma value indicates a more vibrant color, while a lower value signifies a duller or more neutral hue. The hue angle provides information on the perceived color based on its position on the color wheel (0 = red-purple, 90 = yellow, 180 = bluish-green, and 270 = blue) and can be calculated using Equation (2) [40,41]. All experiments were carried out in triplicate, and the results were expressed as the mean value ± SD.

(1) $$C*=\sqrt{(a{*}^{2}+b{*}^{2})}$$

(2) $$\mathrm{hue}=\arctan\left(\frac{b*}{a*}\right)$$

#### 2.4.3. Preparation of Hydroalcoholic Extracts of Yogurt Samples

Yogurt samples were mixed with 45% ethanol in a ratio 1:3 (*w*/*v*). The mixture was heated to 40 °C on a magnetic stirrer, for one hour. Subsequently, the samples were centrifuged (Hettich EBA 21 centrifuge; Andreas Hettich GmbH & Co. KG, Tuttlingen, Germany) at 6000 rpm for 10 min, and the supernatant was collected and stored at 4 °C for 24 h. Finally, the samples were centrifuged once more (6000 rpm, 10 min), and the supernatant was used as the working extract for the analyses in Section 2.4.4, Section 2.4.5, Section 2.4.6.

#### 2.4.4. Total Phenolic Content (TPC)

The TPC analysis of the yogurts was conducted at 0, 7, 14, and 21 days. The TPC of the EDB extract and the hydroalcoholic extracts from the yogurts was quantified using a spectrophotometric method with the Folin–Ciocalteu reagent [42]. Briefly, 100 µL of resolubilized EDB or hydroalcoholic yogurt extract was mixed with 6 mL of distilled water and 500 µL of Folin–Ciocalteu reagent. The mixture was homogenized and incubated for 8 min in the dark. Subsequently, 1.5 mL of Na_2_CO_3_ (7.5%) and 1.9 mL of distilled water were added. The mixture was left for 2 h in the dark at room temperature, and its absorbance was measured at 765 nm using a UV–Vis spectrophotometer (SP-UV 1000 DLAB, Beijing, China). Gallic acid was used as a standard (R^2^ = 0.9995), and the results were expressed as milligrams of gallic acid equivalent (GAE) per gram of lyophilized extract or per gram of yogurt. All experiments were carried out in triplicate, and the results were expressed as the mean value of mg GAE/g ± SD.

#### 2.4.5. Total Flavonoid Content (TFC)

TFC was measured for both the EDB extract and hydroalcoholic extracts of the yogurts. The TFC analysis of the yogurts was conducted at 0, 7, 14, and 21 days. Quantification was performed using a spectrophotometric method described by Pękal and Pyrzynska [43], with some modifications. Here, 2 mL of diluted sample was added to 2 mL of 10% CH_3_COONa and homogenized, after which 2 mL of 2.5% AlCl_3_ was added. The mixture was homogenized and incubated for 60 min in the dark. Absorbance was measured at a wavelength of 420 nm, using a control sample in which the sample was replaced with 45% ethanol. Rutin (R^2^ = 0.9989) was used as the standard solution. All experiments were carried out in triplicate, and the results were expressed as the mean value of milligrams of rutin equivalents (RUE) per gram of lyophilized extract or per gram of yogurt ± SD.

#### 2.4.6. Free Radical-Scavenging Activity

The Free Radical-Scavenging Activity was measured for both the EDB extract and hydroalcoholic extracts of the yogurts. The analysis of the yogurts was conducted at 0, 7, 14, and 21 days. The assay was performed using the free radical DPPH (2,2-diphenyl-1-picrylhydrazyl), according to the method described by Prior et al. [44], with some modifications. The protocol was applied to analyze both the EDB extract and hydroalcoholic extracts of the yogurts. First, a DPPH solution was prepared in 80% ethanol. The sample (0.3 mL) was mixed with 2.7 mL of the DPPH solution, and the mixture was incubated for 60 min in the dark. Absorbance was measured spectrophotometrically at 517 nm. The blank was prepared with 80% ethanol, while the negative control consisted of the DPPH solution mixed with 0.3 mL of 80% ethanol. All experiments were performed in triplicate, and the results are presented as the mean IC_50_ value (mg of EDB extract required to neutralize 50% of free radicals per mL of solution) ± SD. The IC_50_ was determined by plotting the percentage of DPPH inhibition against sample concentration and calculating the corresponding concentration for 50% inhibition using linear regression. RSA% (radical scavenging activity) was calculated using Equation (3),

(3) $$\%\;RSA=\left(1-\frac{{ABS}_{sample}}{{ABS}_{negative\;control}}\right)\times 100$$

### 2.5. Microbiological Analysis

The microbiological quality of the yogurts was evaluated on days 0, 7, 14, and 21. Sample preparation involved mixing 10 g of yogurt sample with 90 mL of sterile Ringer’s solution and homogenizing it using a vortex mixer, resulting in a 10^−1^ g/mL dilution. Subsequently, serial dilutions were prepared to test different microorganisms.

For lactobacilli counting, 1 mL of the diluted sample was placed in a Petri dish and mixed with MRS Agar. To ensure anaerobic conditions, a second layer of medium was applied, and the plates were sealed with parafilm before incubation at 30 °C for 72 h [45].

For mesophilic aerobic bacteria, Plate Count Agar (PCA) was pre-poured into sterile Petri dishes. A volume of 1 mL of the diluted sample was added to each plate, followed by the addition of molten PCA cooled to approximately 45 °C, using the pour plate technique. The plates were then allowed to solidify and incubated at 30 °C for 72 h. After the incubation period, bacterial colony development was observed to assess the analyzed samples.

For psychrophilic bacteria, PCA was pre-poured into sterile Petri dishes. A volume of 100 µL of the diluted sample was evenly spread onto the surface using a sterile Drigalski spatula. The plates were incubated at 7–10 °C for 7 days. After incubation, bacterial colony growth was assessed to determine the psychrophilic microbial load.

The SimPlate kit (Biocontrol^®^) was used to determine coliforms, following the manufacturer’s specifications. Total coliforms were identified by a color change from blue to pink, while quantification was performed by counting wells exhibiting fluorescence under UV light (365 nm) [37].

For yeast and mold quantification, DRBC Agar was used according to the manufacturer’s instructions [46]. Growth was monitored until day 7 of incubation at 25 °C.

All the results were expressed as the mean value of logarithmic colony-forming units (CFU) per gram of yogurt ± SD.

### 2.6. Sensorial Analysis

For the samples obtained after carrying out the entire process (OYC, OY1, OY2, OY3, and Oyc), 41 untrained panelists were randomly selected from among students and staff at Instituto Politécnico de Bragança, Portugal. For the samples obtained in Romania (CYC, CY1, CY2, and CY3), 53 untrained panelists were randomly selected from among students and staff at the University of Agronomic Sciences and Veterinary Medicine of Bucharest, Romania.

The homogenized yogurt samples were distributed in matte white glasses, with approximately 30 g of yogurt per sample per panelist. For each sample a three-digit code was assigned and they were analyzed in a random order. The organoleptic characteristics of the yogurts (aroma, color, flavor, and consistency) were evaluated on a scale from 1 to 5, where 1 represented the lowest score for the evaluated characteristic and 5 the highest one. Panelists provided their evaluations based on personal preference, which may have influenced their scoring of the different characteristics. The evaluation criteria were as follows.

Aroma: 1—No aroma/Unpleasant aroma; 2—Weak aroma; 3—Moderate aroma, pleasant but not strong; 4—Pleasant, intense, well-balanced aroma; 5—Very intense, highly pleasant aroma.

Color: 1—Non-existent/Unpleasant color; 2—Weak color, pleasant; 3—Moderate, pleasant, and uniform color; 4—Intense, pleasant, and uniform color; 5—Very intense, attractive, and uniform color.

Flavor: 1—No flavor/Unpleasant flavor; 2—Weak flavor; 3—Moderate flavor, pleasant but not strong; 4—Pleasant, intense, well-balanced flavor; 5—Very intense, pleasant, and highly appreciated flavor.

Consistency: 1—Watery, too thin/Unpleasant consistency; 2—Thin consistency, slightly unpleasant; 3—Moderate, typical for yogurt, pleasant; 4—Creamy, pleasant consistency; 5—Very smooth, creamy, and pleasant.

Panelists were also asked to select their preferred sample and indicate whether they would consider purchasing it. To maintain the sensitivity and accuracy of taste and olfactory evaluations while preventing cumulative effects and interference between successive evaluations, panelists were provided with room-temperature water and apple slices as palate cleansers. Participants were informed about the sensory evaluation procedure before its commencement. The sensory evaluation study was approved by the Ethics Committee of the University of Agronomic Sciences and Veterinary Medicine of Bucharest, Romania.

### 2.7. Statistical Analysis

All analyses were performed in triplicate, and statistical significance was set at *p* < 0.05 and *p* < 0.01. The results were analyzed using the IBM SPSS Statistics software, version 29 (IBM Corp., Armonk, NY, USA). Comparisons between samples were tested by one-way ANOVA, followed by Student’s t-test for comparisons with control samples. Duncan’s Multiple Range Test (DMRT) was applied to separate the means at a *p* < 0.05 significant difference. Data were expressed as means ± SD.

## 3. Results and Discussion

### 3.1. Chemical Analysis of EDB Extract

EDBs contain significant amounts of phenolic compounds, as demonstrated by the analysis of the fruit extract used in yogurt production, with 6369.52 ± 32.99 mg GAE/100 g lyophilized extract. This finding is consistent with numerous other studies [47,48]. Flavonoids represent one of the most important classes of phenolic compounds in EDBs, with a quantified content of 1271.63 ± 17.30 mg RUE/100 g lyophilized extract. The main flavonoids are anthocyanins, such as cyanidin-3-glucoside and cyanidin-3-sambubioside, which contribute to the fruits’ intense coloration and exhibit notable antioxidant properties [49]. Analysis of the antioxidant activity of the fruit extract showed that it produced an inhibition corresponding to an IC_50_ value (the concentration required to neutralize 50% of free radicals) of 0.678 mg/mL, while for ascorbic acid it was 0.034 mg/mL. Duymuş et al. [50] also demonstrated that EDB extracts exhibit antiradical activity against DPPH, although not as potent as ascorbic acid, which was used as a reference. The IC_50_ values were lower than those in our study: 0.117 mg/mL for the 70% acetone extract and 0.123 mg/mL for the water extract of EDB. In comparison, ascorbic acid exhibited a much lower IC_50_ value of 0.080 mg/mL, indicating a stronger antioxidant capacity.

The study conducted by Haș et al. [51] demonstrated that EDB from the spontaneous flora of Romania possesses notable antioxidant and antimicrobial properties. In this study, freeze-dried EDB powder was evaluated at concentrations of 0.5%, 1%, and 1.5%, with a focus on the interplay between bioaccessibility, prebiotic effects, and the overall health benefits provided by bioactive compounds [52]. The results reveal 74.54% TPC bioaccessibility, 60.64% for flavonols, and 53.17% for anthocyanins, suggesting their potential to exert prebiotic effects, a finding supported by a previous study [53]. The relationship between polyphenols and intestinal microbiota appears to be bidirectional—phenolic compounds can modulate the intestinal microbiota, while microorganisms can also influence polyphenol activity [54]. Based on the results obtained and the data available in the scientific literature, adding EDB extract to yogurt could be an effective way to incorporate these bioactive compound-rich fruits into the daily diet.

### 3.2. Investigation on Samples from Homemade Yogurt

#### 3.2.1. Physicochemical Analysis of Homemade Yogurt Samples

The pH analysis results are presented in Table 1. At the beginning, pH values showed slight variations between the samples, with average pH ranging from 4.17 to 4.24, indicating moderate acidity, characteristic for fresh yogurt. The addition of EDB extract led to a slight increase in pH across the samples. By day 7, a minor decrease in pH was observed (*p* > 0.05), suggesting continued fermentation, albeit at a slower rate compared to higher temperature conditions. By day 14, most samples showed a further decline in pH, with the control samples showing more stable values (*p* < 0.05). A more pronounced pH decrease was seen in OY2, OY3, and OY4 (*p* < 0.05), indicating that even a small percentage of EDB extract influenced the fermentation process. In some cases, lactic acid degradation or its utilization by non-acidifying microorganisms, such as contaminating bacteria or yeasts, may have contributed to this pH increase, thus reducing overall acidity. The observed statistically significant increase in pH in the control yogurt sample over the 21-day storage period could be attributed to the growth of psychrophilic bacteria, yeasts, and molds. The microbiological results, presented later, suggest that the microbial growth, which was present only in the control sample throughout the entire storage period, may have contributed to a decrease in acidity, thereby leading to the observed increase in pH. This underscores the importance of pH monitoring in yogurts as a critical factor for ensuring product safety [55].

Chouchouli et al. [56] investigated the effect of polyphenols on yogurt by using yogurts with varying fat contents (full-fat and non-fat) and grape seed extracts. They observed that the addition of the grape seed extracts did not affect the pH, and no differences were found between full-fat and non-fat yogurts.

One of the most important sensory factors affecting consumer choice is color, which also affects the product’s overall identification and appearance. The color characteristics of the samples (CIE L*a*b*, C*, and °hue values) are shown in Table 2. Overall, there were significant differences in color parameters (*p* < 0.01) between the control samples and those with EDB extract. OYC and OY1 exhibited higher lightness (L*), indicating a relatively light-colored yogurt. The predominant color in these samples was yellow-green, with a low saturation (low C*), suggesting a relatively pale yogurt, as no natural dyes or extracts were added. The sample OY2 showed a lower L* value, indicating a darker color compared to the control sample (*p* < 0.05). The a* and b* values suggest a reddish tint, probably due to the EDB extract, which contributed a different hue while still maintaining a relatively pale appearence. The OY3 sample had a lower L* value, indicating an even darker color compared to OY2 (*p* < 0.05). The hue shifted towards red and the C* continued to increase, indicating that a higher concentration of EDB extract resulted in a more noticeable color change. Sample OY4 had the lowest L* value, indicating it was significantly darker (*p* < 0.01) than others. The C* value for this sample indicates that the color intensified with an increasing percentage of the extract, predominately shifting towards shades.

**Figure 2:**
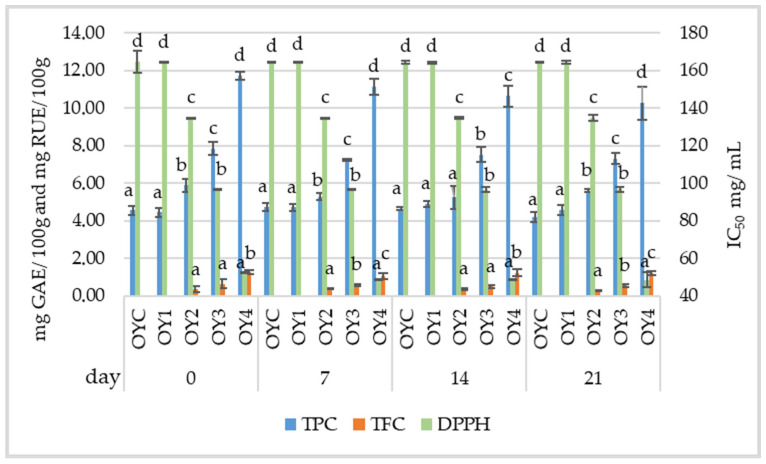
Changes in total phenolic content (TPC), total flavonoid content (TFC), and IC_50_ concentration in samples obtained from homemade yogurt at 0, 7, 14, and 21 days of storage. The data are presented as mean values (*n* = 3) ± standard deviation. Different superscript letters within the same parameter indicate significant differences between the experimental variants (*p* < 0.05) at each time point. OYC = control yogurt; OY1 = yogurt with 0.1% potassium sorbate; OY2 = yogurt with 0.05% potassium sorbate and 0.05% EDB extract; OY3 = yogurt with 0.1% EDB extract; OY4 = yogurt with 0.2% EDB extract.

Following the analysis of yogurt samples with different concentrations of EDB extract and potassium sorbate over 21 days, significant trends were observed regarding the TPC and TFC (Figure 2). The samples containing EDB extract (OY2, OY3, OY4) had higher TPC and TFC values compared to OYC and OY1, indicating that the addition of EDB extract in the yogurt formulation significantly (*p* < 0.01) contributes to an increase in bioactive compounds. The presence of TPC in the control samples may be attributed to the interference of other compounds within the sample, which could interact with the Folin–Ciocalteu reagent, such as proteins, peptides, amino acids, unsaturated fatty acids, carbohydrates, organic acids, and inorganic ions [57]. OY4 recorded the highest initial TPC value of 11.71 ± 0.22 mg GAE/100 g, which decreased to 10.26 ± 0.89 mg GAE/100 g by day 21. This decrease suggests a slight degradation of polyphenolic compounds during storage. Kim et al. [58] reported an increasing trend in TPC during the 7-day storage of yogurt, both with and without lotus leaf. This increase was attributed to the proteolysis of milk proteins, which released amino acids containing phenolic side chains. Additionally, the authors suggested that microbial metabolism might have led to the formation of new phenolic acids, further contributing to the rise in total polyphenol levels. Anuyahong et al. [59] formulated a yogurt by incorporating rice-berry (*Oryza sativa* L.), a purple pigmented rice variety from Thailand, in the form of extract at concentrations ranging from 0.125% to 0.5% *w*/*w*. Their results show that the addition of rice-berry extract led to a significant increase in the yogurt’s phenolic content, along with higher levels of peonidin-3-glucoside, cyanidin-3-glucoside, and anthocyanins, without negatively affecting the yogurt’s quality parameters.

The presence of TFC was recorded in the cases of OY2, OY3, and OY4 samples, with the values remaining stable throughout the 21-day period. OY4 had the highest value (*p* < 0.01), starting at 1.26 ± 0.12 mg RUE/100 g on day 0, with the value slightly decreasing to 1.21 ± 0.10 mg RUE/100 g by day 21. OY3 and OY2 exhibited relatively lower, but still detectable, flavonoid levels. These results suggest that the EDB extract, even in small concentrations, contributes to the TFC of the yogurt, although degradation or oxidation occurs over time. In contrast, flavonoids were not detected (ND) in OYC and OY1. This absence highlights the key role of the EDB extract in enhancing the flavonoid profile of the yogurt. The samples with EDB extract (OY2, OY3, OY4) reached their IC_50_ at lower concentrations than the control samples (*p* < 0.01), suggesting that EDB extract has a much stronger inhibitory effect, being effective even at low doses. Bourassa et al. [60] investigated the influence of α-casein on the antioxidant activity of tea phenolic compounds. The results show that the milk protein had a minor impact on the antioxidant capacity of polyphenols. Caleja et al. [55] investigated the use of chamomile and fennel decoctions as natural additives in yogurt, comparing their effects to potassium sorbate. Their findings show that yogurts fortified with potassium sorbate exhibited a more rapid decline in antioxidant capacity compared to those containing plant-based additives, particularly from the seventh day of storage onward. This suggests that plant extracts may offer better protection against oxidative degradation, potentially due to their complex composition of bioactive compounds with synergistic effects. Hong et al. [61] demonstrated that the addition of freeze-dried safflower (*Carthamus tinctorius* L.) at concentrations of 0.1%, 0.5%, and 1.0% in yogurt led to an increase in protein, fat, lactose, TPC, TFC, and antioxidant activity. Additionally, the study reported a decrease in moisture content and pH values.

Proteins in yogurt play a crucial role in maintaining the integrity of phenolic compounds during digestion, enhancing their bioaccessibility [62]. Additionally, yogurt proves to be an excellent delivery vehicle for phenolic compounds from plant extracts, as its low pH (4.1–4.5) helps preserve their stability during storage [58,63]. However, further research on the bioavailability of these compounds is necessary to fully understand their absorption, metabolism, and potential health benefits.

#### 3.2.2. Microbiological Analysis of Homemade Yogurt Samples

Homemade yogurt was prepared using commercial yogurt as the inoculum. Although this approach did not allow control over specific bacterial strains, it ensured a consistent microbial community by utilizing a commercially available product with known fermentation characteristics. The objective of this phase was not to control the microbial species but to evaluate the impact of EDB extract on the microbiological, chemical, and sensory properties of yogurt, while maintaining the viability of beneficial microorganisms. This part of the study was focused on two types of bacteria: lactic acid bacteria (LAB), responsible for yogurt fermentation and the development of its texture and flavor, and mesophilic bacteria, which are not essential for fermentation but may influence the product’s shelf life and overall quality. It has been recommended that foods containing probiotic bacteria should meet the suggested minimum value of 10^6^ CFU/g to achieve optimal potential therapeutic effects [64]. The main microbiological goal was to preserve LAB, which are crucial for the key qualities of the product, while also monitoring mesophilic bacteria to assess their impacts on yogurt quality during storage. The analysis of LAB over 21 days revealed important trends related to the preservation and growth of beneficial microorganisms in yogurt (Table 3). The initial count of LAB was similar across all samples, with values ranging from 8.69 to 8.82 log CFU/g. This indicates that the presence of potassium sorbate and EDB extract did not significantly affect the growth of LAB in the early stages. At day 7, the analysis showed a slight decline in LAB count, suggesting that the LAB remained active. A more noticeable reduction occurred after 14 days. OYC, the control sample, showed the smallest decline (7.60 ± 0.10 log CFU/g), while the yogurt with potassium sorbate (OY1) decreased even more (6.39 ± 0.09 log CFU/g). The samples containing EDB extract (OY2, OY3, and OY4) showed a smaller reduction compared to OY1, suggesting that the EDB extract might have provided some degree of protection to the LAB. However, this effect was not sufficient to fully preserve their viability. The final day revealed the greatest reduction in LAB counts, with sample OY2 showing the lowest count (5.34 ± 0.04 log CFU/g; a loss of 3.35 log CFU/g). This suggests that the combination of potassium sorbate and EDB extract, especially at this concentration, had a more pronounced negative effect on LAB over time. Samples OY3 and OY4 showed relatively higher counts, suggesting that higher concentrations of EDB extract may be more effective in preserving LAB, though they did not prevent the overall decline. These findings align with the results reported by Ranadheera et al. [65], who observed a significant decrease in *Lactobacillus delbrueckii* subsp. *bulgaricus* counts during storage. By comparison, *L. delbrueckii* subsp. *bulgaricus* counts in stirred fruit yogurts decreased markedly in the fourth week, from nearly 10⁸ CFU/g to approximately 10⁵ CFU/g, while in plain yogurts, they remained at around 10⁶ CFU/g. In contrast, Vinderola et al. [66] observed no significant changes in *L. delbrueckii* subsp. *bulgaricus* counts in plain yogurt after 28 days of storage at 5 °C. These findings highlight the importance of carefully selecting probiotic strains and starter cultures when formulating different types of yogurt to ensure microbial stability over time.

For mesophilic bacteria, all yogurt samples exhibited an increase in bacterial counts during the first 7 days of storage. This initial growth is a common adaptation response of microorganisms to their environment. However, starting from day 14, bacterial counts began to decline, continuing through day 21, which aligns with the expected natural decrease as nutrients are depleted and preservatives take effect. This trend is consistent with the known antimicrobial properties of potassium sorbate, which progressively inhibit bacterial growth during storage [67]. The EDB extract combined with sorbate starts to show its inhibitory effects after the first 7 days. OY3 and OY4 followed similar patterns, with bacterial counts decreasing more significantly at later stages of storage. On day 7, OY3 (7.59 ± 0.07 log CFU/g) and OY4 (7.32 ± 0.02 log CFU/g) exhibited higher bacterial counts compared to OY2 (6.35 ± 0.04 log CFU/g), but by day 21, the decrease in bacterial counts was evident (5.67 ± 0.05 CFU/g for OY2, 5.23 ± 0.07 log CFU/g for OY3, and 4.34 ± 0.04 log CFU/g for OY4).

These results suggest that the EDB extract combined with potassium sorbate exerts an inhibitory effect during the initial storage period, while the simple EDB extract leads to a gradual decline in bacterial counts, indicating a delayed antimicrobial action. This could be attributed to the interaction between the extract and microbial cells, as well as the accumulation of the extract’s active compounds in the yogurt over time. Although the number of probiotic bacteria decreased during storage, probiotic yogurts maintained probiotic properties until the end of the storage period, highlighting their potential health benefits.

During storage, the development of psychrophilic bacteria was strongly influenced by the type and concentration of additives. On day 0, OYC exhibited a value of 2.26 ± 0.06 log CFU/g, while in the other samples (OY2, OY3, OY4), psychrophilic bacteria were <1 CFU/g. OY2 also showed a small initial presence of psychrophilic bacteria at 1.60 ± 0.03 log CFU/g, suggesting that the combination of potassium sorbate and EDB extract was not fully effective in preventing their initial presence at the beginning of storage. As storage time progressed, OYC showed a slight increase in the number of psychrophilic bacteria, reaching 2.99 ± 0.07 log CFU/g on day 14 and 3.71 ± 0.10 log CFU/g on day 21. In contrast, in samples OY1, OY2, OY3, and OY4, the number of psychrophilic bacteria remained <1 CFU/g even by the end of the storage period. This result suggests that the additives in these samples were highly effective in inhibiting the growth of psychrophilic bacteria, which could be advantageous for prolonging shelf life while ensuring product quality and safety during storage.

The analysis of yeasts and molds during the storage of the yogurts reflects the overall effectiveness of the preservatives in controlling microbial growth. The yogurt was prepared using UHT milk, which theoretically should be free of yeasts and molds due to high-temperature processing. Furthermore, the preparation was conducted under sterile conditions to minimize contamination risks. However, the incorporation of powdered milk and yogurt inoculation may have introduced a minor risk of contamination. On day 0, OYC contained 1.72 ± 0.06 log CFU/g of yeasts and molds, while all the other samples (OY1, OY2, OY3, OY4) showed <1 CFU/g, indicating that the additives, particularly potassium sorbate and EDB extract, were initially effective in preventing yeast and mold contamination in the yogurt. Throughout storage, yeast and mold levels in OYC progressively increased, reaching 3.32 ± 0.07 log CFU/g by day 21. This suggests that the simple yogurt (OYC) experienced microbial growth during the storage period, likely due to the absence of preservatives that would otherwise inhibit this type of contamination. For the samples containing potassium sorbate and EDB extract, whether used individually or in combination (OY1, OY2, OY3, OY4), yeasts and molds remained <1 CFU/g. Potassium sorbate is widely used in yogurt production to inhibit mold and yeast growth, thereby extending its shelf life. Research suggests that potassium sorbate can also slow post-acidification induced by starter cultures like *S. thermophilus* and *L. bulgaricus*. The concentrations commonly tested for its application are 0.005%, 0.05%, and 0.1% (*w*/*v*). At these concentrations, potassium sorbate effectively reduces acid production and microbial activity throughout storage while preserving the sensory properties of yogurt [67,68]. EDB extract has the potential to serve as an alternative to potassium sorbate in yogurt production, ensuring the safety of the product during storage by inhibiting microbial growth. Higher concentrations of EDB extract do not seem to affect the viability of LAB, thus maintaining the probiotic benefits of yogurt upon consumption. Nonetheless, further research is required to assess its long-term impacts on yogurt’s functional properties and to explore its broader applicability as a food preservative in various formulations.

#### 3.2.3. Sensorial Analysis of Homemade Yogurt Samples

**Figure 3:**
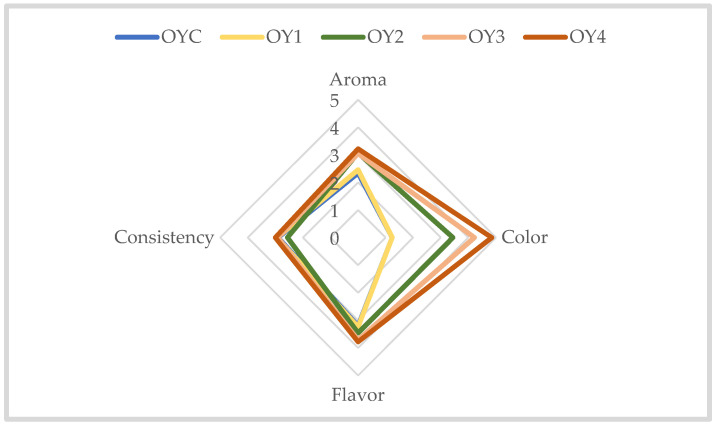
Sensory profile of samples obtained from homemade yogurt: aroma, color, flavor, and consistency. OYC = control yogurt; OY1 = yogurt with 0.1% potassium sorbate; OY2 = yogurt with 0.05% potassium sorbate and 0.05% EDB extract; OY3 = yogurt with 0.1% EDB extract; OY4 = yogurt with 0.2% EDB extract.

Since freeze-dried plant extracts with larger particles (>25 microns) often cause a sandy mouthfeel [69], the extract was first solubilized before being added to the yogurt, in order to avoid affecting its sensory characteristics. The organoleptic evaluation, which assessed aroma, color, flavor, and consistency, revealed notable differences among the yogurt samples (Figure 3). The aroma was generally moderate across all samples, with OY4 receiving the highest score (3.22 ± 1.15), followed by OY3 (3.05 ± 1.59), suggesting that the addition of EDB extract resulted in a more pleasant scent compared to OYC (2.34 ± 0.93) or OY1 (2.46 ± 1.14). The initial objective color analysis revealed a significant increase in intensity with the addition of EDB extract (*p* < 0.01), likely due to the natural pigments present in the extract. This observation was further supported by the sensory analysis, where OY4 achieved the highest score for color intensity (4.85 ± 0.85), reflecting its intense, pleasant, and uniform color, as preferred by the panelists. These findings indicate that the enhanced color intensity observed in the objective analysis was not only measurable, but also highly appreciated by the panel. Ren and Chen [70] reported that product color influences 62–90% of purchasing decisions.

Regarding flavor, the samples OY4 (3.78 ± 1.15) and OY3 (3.71 ± 1.14) were rated higher than sample OY1 (3.24 ± 0.98) or OYC (3.20 ± 0.96), indicating that the extract contributed to a more appealing taste. The consistency remained relatively similar across all samples, with a slight improvement in OY4 (3.00 ± 0.92) compared to OYC, OY1, OY2, and OY3, which may be attributed to the extract’s influence.

In terms of overall acceptability, participants favored the yogurt samples enriched with EDB extract, with 68% favoring OY3 (34%) or OY4 (34%), compared to OYC (10%), OY1 (12%), and OY2 (10%). Additionally, 82.93% of individuals indicated that they would purchase their most preferred sample, highlighting the potential consumer appeal of EDB extract as a functional ingredient in yogurt production.

Roy et al. [71] found that papaya yoghurt was the most preferred among variants with watermelon, papaya, and banana. Angelov et al. [72] reported that mulberry-flavored yoghurt was favored over molasses, while Tarakci and Kucukoner [73] observed a preference for yoghurts with sour cherries and grape molasses over those with dates, rosehips, or cranberries. These studies highlight the influence of fruit selection on yoghurt acceptability, supporting research on elderberry-enriched yoghurts. Findings indicate that at low concentrations of EDB extract (0.1% or 0.2%), polyphenols do not negatively impact sensory properties, and may even enhance overall acceptance, reinforcing the potential of bioactive compounds to improve both taste and nutritional value.

### 3.3. Investigation on Samples from Commercial Yogurt

The results obtained from the first experiment (testing the OYC, OY1, OY2, OY3, and OY4 samples) indicate that the incorporation of EDB extract into yogurt enhances its content of health-promoting compounds while preserving probiotic activity and product stability over time. However, the addition of the extract at concentrations of 0.05%, 0.1%, and 0.2% showed limited antioxidant activity. In the second experiment, we aimed to enhance the bioactive compound concentration in the yogurt by adding 0.5% elderberry extract, while also testing the effect of incorporating a sweetener.

Numerous polyphenolic compounds exhibit an unpleasant taste, primarily characterized by astringency and bitterness, which characteristics are mainly induced by flavonol polymers such as proanthocyanidins or condensed tannins [74]. These undesirable flavors must be masked before the compounds are incorporated into food products or oral medications [75]. In addition, a strategy was employed to enhance product acceptability by incorporating a natural sweetener, agave syrup. Sucrose can be added in proportions of up to 6% (*v*/*w*) in fruit yogurts [40]. Since agave syrup has a higher sugar content than many syrups rich in glucose or sucrose—such as honey and maple syrup—a lower amount is required to achieve the desired sweetness (4% *v*/*w*), resulting in reduced caloric intake [76].

#### 3.3.1. Physicochemical Analysis of Commercial Yogurt Samples

In the experiment, similar pH trends were observed across all yogurt samples analyzed during the 21-day storage period (Table 4). For the samples CYC and CY1, a similar pattern was observed, with an increase up to day 14, followed by a slight decrease on day 21. For samples CY2 and CY3, the pH increased during the first 14 days, followed by a slight decrease on day 21. This trend suggests that the EDB extract did not have a significant effect on acidification. Considering that the base yogurt was commercially produced and already fermented and stabilized, the observed pH changes during storage were minimal. The controlled fermentation environment of the commercial yogurt might explain the relatively small fluctuations in pH observed during the storage period. Similarly, Abdel-Hamid et al. [77] showed that the incorporation of 0.5% *Siraitia grosvenorii* fruit extract into probiotic yogurt did not cause a significant (*p* > 0.05) alteration in pH when compared to the control sample. In contrast, Yousef et al. [78] reported that strawberry yoghurt had a lower pH value than plain yoghurt, suggesting that the addition of fruit or other ingredients can lead to greater variability in pH, depending on the composition and fermentation conditions.

The color characteristics show that CYC and CY1 had very similar chromatic profiles (Table 5). Both exhibited high luminosity, moderate a* and b* components, and a stable hue, indicating a neutral and balanced color. The addition of syrup in CY1 did not result in significant changes compared to CYC (*p* > 0.05), meaning that the agave syrup did not notably affect the color profile of the sample. The samples with extract (CY2 and CY3) showed more pronounced changes in color compared to CYC and CY1. Specifically, CY2 displayed a significantly lower L* (*p* < 0.01), indicating a darker color. The a* component was much higher, suggesting a stronger red hue (*p* < 0.01), while the b* component was much lower, indicating a cooler tone. CY3 had a similar trend, with even more pronounced redness (a* component) and a slightly warmer hue compared to CY2. Both samples showed significant differences compared to the controls (*p* < 0.01).

**Figure 4:**
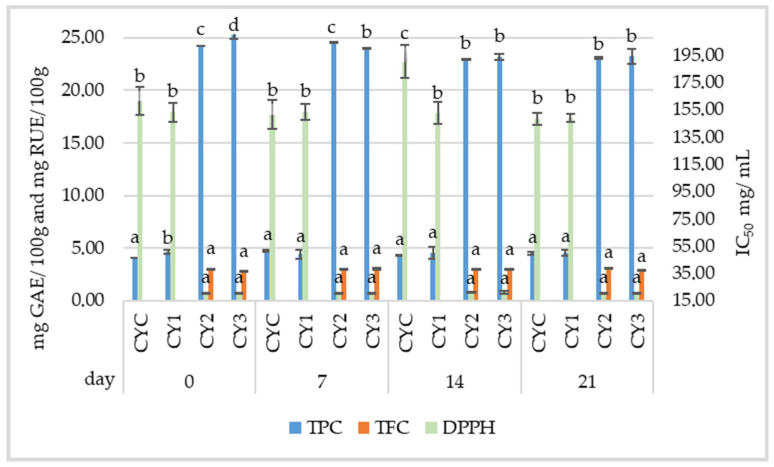
Changes in total phenolic content (TPC), total flavonoid content (TFC), and IC_50_ concentration in samples obtained from commercial yogurt on 0, 7, 14, and 21 days of storage. The data are presented as mean values (*n* = 3) ± standard deviation. Different superscript letters within the same parameter indicate significant differences between the experimental variants (*p* < 0.05) at each time point. CYC = control yogurt; CY1 = yogurt with 4% agave syrup; CY2 = yogurt with 0.5% EDB extract; CY3 = yogurt with 0.5% EDB extract and 4% agave syrup.

The TPC values were significantly higher in the yogurt samples containing EDB extract (CY2 and CY3) compared to CYC and CY1 throughout the 21-day storage period (Figure 4). On day 0, CY2 and CY3 exhibited considerably higher TPC values (24.26 ± 0.17 mg GAE/100 g and 25.29 ± 0.38 mg GAE/100 g, respectively) compared to the control (4.11 ± 0.00 mg GAE/100 g) or sample CY1 (4.61 ± 0.00 mg GAE/100 g). These values remained relatively stable over the storage period. The TFC was detected in yogurt samples with EDB extract, CY2 and CY3, containing 3.03 ± 0.03 mg RUE/100 g and 2.80 ± 0.04 mg RUE/100 g, respectively, on day 0. Throughout the storage period, TFC values exhibited slight fluctuations but remained significantly higher than those in the control samples, where flavonoids were not detected. These findings suggest that EDB extract significantly enhanced the TPC and TFC of the yogurts, with no degradation of the active compounds throughout the storage period. The addition of agave syrup in the CY3 sample did not significantly impact TPC or TFC (*p* > 0.05), suggesting no interaction with the phenolic or flavonoid compounds. The CY2 and CY3 samples exhibited significant antioxidant activity (*p* < 0.05) throughout the experiment (stable IC_50_ values around 20 ± 0.47 mg/mL) compared with CYC and CY1 samples (151–153 mg/mL).

In contrast to the results of this study, some studies have shown an increase in TPC and antioxidant activity during fermentation. This could be attributed to the hydrolysis of complex phenolic compounds into simpler forms and the formation of antioxidant metabolites during the fermentation process [79]. The study conducted by Durmus et al. [80] demonstrated that adding mulberry fruit before yogurt fermentation resulted in low antioxidant activity, which was attributed to the degradation of anthocyanins during incubation at 43 °C and the interaction of phenolic compounds with proteins or peptides formed during the fermentation process. The antioxidant activities of all samples were further reduced after storage for 21 days. Therefore, the timing of adding the fruits is critical to preserving antioxidant properties and minimizing the loss of bioactive compounds. Herrera et al. [81] demonstrated that wild fruit extracts (strawberry tree and hawthorn) incorporated into yogurt not only enhanced antioxidant properties, but also exhibited antidiabetic effects by inhibiting the enzymatic activities of α-amylase, α-glucosidase, and lipase. These benefits were achieved without negatively affecting the sensory quality or consumer acceptance of the product.

#### 3.3.2. Microbiological Analysis of Commercial Yogurt Samples

Commercial yogurt was used as the base in this experiment to ensure consistency in the yogurt’s microbiota, to simplify the procedure, and to focus on the effects of the EDB extract and agave syrup on the functional and sensory properties of the product. The results indicate that the LAB count remained relatively stable throughout the 21-day storage period, with only a slight decrease observed across all yogurt samples (Table 6). Initially, bacterial counts ranged between 6.30 and 6.46 log CFU/g, declining slightly to values between 5.95 and 6.01 log CFU/g by day 21. This trend aligns with the expected decline of LAB viability over time due to nutrient depletion. It is recommended that yogurts with probiotic benefits contain at least 10^6^ CFU/g of probiotic bacteria at the end of their shelf life in order to make health-related claims [82]. Given that even the control yogurt exhibited low bacterial counts from the beginning, it is possible that storage conditions prior to purchase affected bacterial viability. This highlights the importance of monitoring the cold chain, as well as transport and storage conditions, to ensure the preservation of probiotic properties in dairy products. The addition of EDB and agave syrup did not significantly influence the survival of these bacteria, as the values remained comparable across all samples. Similarly, mesophilic bacteria exhibited a stable trend, with initial values between 6.29 and 6.31 log CFU/g and a slight reduction by day 21 (6.17–6.24 log CFU/g). Abdel-Hamid et al. [77] showed that while the addition of 0.5% *Siraitia grosvenorii* fruit extract had no significant (*p* > 0.05) effect on *Lactobacillus bulgaricus*, the incorporation of 1% and 2% fruit extract significantly (*p* < 0.05) enhanced the viability of *L. bulgaricus*. Therefore, the percentage of fruit extract plays a crucial role in stimulating the growth of probiotic bacteria in yogurt. Varga [83] reported that yoghurt containing 5% honey showed no significant changes in lactic acid development, pH, or microbial growth. Similarly, the minor variations observed over time in this study suggest that fermentation and storage conditions maintained bacterial stability, with no significant impact caused by the presence of EDB extract or agave syrup.

Psychrophilic bacteria, yeasts, molds, and coliforms were not detected in any of the samples throughout the storage period, with counts below the detection limit (<1 CFU/g), confirming the microbiological quality of the yogurt and the effectiveness of storage conditions in preventing contamination or spoilage. The absence of these microorganisms highlights the microbiological quality of the commercial base yogurt and its stability under the given conditions. The stability of LAB and mesophilic bacteria over the 21-day period can also be attributed to the commercial nature of the yogurt, which is formulated to maintain microbial viability. This stability was a key aspect of our investigation—assessing whether the addition of EDB extract and agave syrup would induce significant changes in a product with an already stable microbial profile. The results suggest that these ingredients did not disrupt the natural microbial dynamics of yogurt, indicating their potential for incorporation without compromising microbiological stability.

#### 3.3.3. Sensorial Analysis of Commercial Yogurt Samples

**Figure 5:**
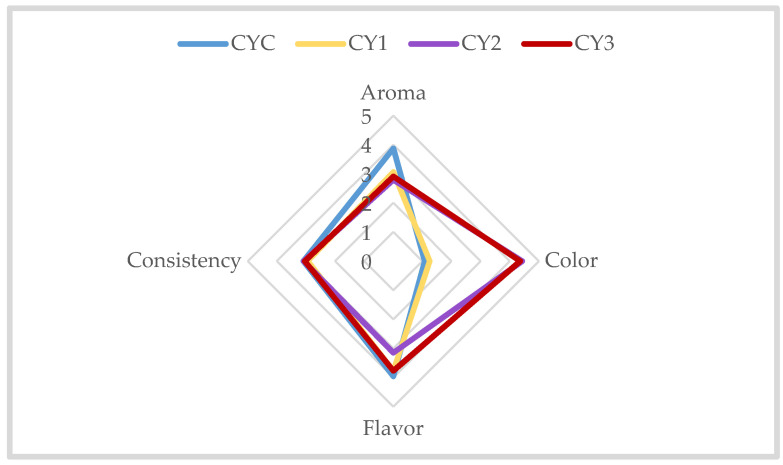
Sensory profile of samples obtained from commercial yogurt: aroma, color, flavor, and consistency. CYC = control yogurt; CY1 = yogurt with 4% agave syrup; CY2 = yogurt with 0.5% EDB extract; CY3 = yogurt with 0.5% EDB extract and 4% agave syrup.

The organoleptic evaluation (Figure 5) showed that aroma was pronounced in CYC (3.87 ± 1.13), and less intense in CY1 (3.06 ± 1.20), CY2 (2.78 ± 1.11), and CY3 (2.91 ± 1.28). The inclusion of agave syrup and EDB extract likely attenuated the characteristic yogurt aroma. The color intensity, as evaluated through color analysis, was highest in CY2 and CY3, which exhibited more vibrant hues, while the control samples showed much paler tones, close to white. These findings align with the sensory analysis, where CY2 and CY3 were preferred for their more intense and attractive color, further emphasizing the influence of EDB extract on color enhancement. Flavor was most intense in CYC (3.96 ± 1.10), which scored close to a pleasant, intense, well-balanced flavor, while CY1 and CY3 both scored 3.77 ± 1.09 and 3.77 ± 0.96, respectively, indicating a more moderate, pleasant but not strong flavor. CY2 (3.15 ± 0.57) exhibited the lowest intensity, corresponding to a flavor that was pleasant but mild. The strong astringent effect of free polyphenols may also negatively influence the flavors of various foods [84], which could explain the results obtained for the sample with extract and without sweetener. Consistency was relatively similar across all samples, with CYC (3.09 ± 0.89) showing a slightly firmer texture (*p* > 0.05).

In terms of overall acceptability, participants favored the yogurt samples enriched with EDB extract and agave syrup, with 49.06% expressing a preference for CY3, 26.41% for CY1, 22.64% for CYC, and 1.89% for CY2. Additionally, 100% of participants indicated they would purchase CY3, the most preferred sample based on overall acceptability.

Since fermented dairy products have a distinctive sour taste, which may negatively influence consumer acceptability [85], the association between flavor and color can also play a crucial role in perception. Dark-colored fruits, such as elderberries, are often linked to a sweet or mildly tart taste rather than the pronounced sourness of fermented dairy. This mismatch in expectations may explain why consumers preferred familiar options, such as plain yogurt or the sample sweetened with EDB extract and agave syrup. A noticeable reluctance for new flavors was observed (as in the case of CY2); however, the addition of agave syrup improved the product’s palatability by masking the unpleasant taste of polyphenols, making the CY3 sample the most preferred option. Similar results were reported by Herrera et al. [81], who found that the control yogurt obtained a significantly higher overall liking score (*p* < 0.05) compared to yogurts containing hawthorn extracts. This high acceptance of the control yogurt could be attributed to consumers’ familiarity with conventional yogurt, reinforcing the preference for well-known flavors over novel formulations.

Barazi et al. [86] studied the enhancement of kefir functionality by adding black EDB. The results show that the general appreciation scores were above 3 on a 5-point hedonic scale. Notably, the samples produced with dried EDB powder (0.5% and 1% dried EDB powder) were more highly appreciated compared to those made with fresh EDB (2% and 4% heat-treated fresh EDB fruit mash). Du and Myracle [30] formulated EDB kefir using 4.3% or 5.7% sucrose. Additionally, they used 0.4% and 0.6% stevia extracts to achieve sweetness equivalent to the sucrose-based formulations. The kefir sweetened with 5.7% sucrose had the highest acceptability in terms of sweetness. In contrast, products containing stevia extract received significantly lower ratings, likely due to its unpleasant aftertaste, as noted by consumers. This supports the use of agave syrup as a sweetener, as observed in this study, where it did not introduce an undesirable aftertaste. Therefore, agave syrup can serve as a viable alternative for maintaining sensory balance in fermented dairy products.

## 4. Conclusions

This study investigated the effects of EDB extract, potassium sorbate, and agave syrup on the physicochemical, microbiological, and sensory properties of yogurt. In the first experiment, yogurt samples with varying concentrations of EDB extract and potassium sorbate demonstrated significant improvements in antioxidant activity, as indicated by increased TPC and TFC. Microbiological analysis showed minimal microbial contamination across all samples, with no detection of coliform bacteria, and this stability was maintained throughout the storage period, suggesting the possibility of replacing potassium sorbate. Sensory evaluation revealed that higher concentrations of EDB extract enhanced both flavor intensity and color, with the sample containing the highest concentration of extract being the most preferred.

In the second experiment, the inclusion of 0.5% EDB extract enhanced the bioactive compound content, potentially contributing to the health benefits of the yogurt. Microbiological stability was maintained throughout the study, with no significant microbial growth observed, ensuring the safety and quality of the yogurt samples. The combination of EDB extract and agave syrup positively influenced sensory attributes such as flavor, color intensity, and texture, with the sample containing both ingredients being the most preferred by panelists.

Future studies could focus on the potential health benefits of EDB extract and agave syrup in yogurt, particularly their long-term effects on nutritional value, functional properties, and gut microbiota. It is also essential to validate the in vivo health benefits of these ingredients and assess consumer acceptance in a larger population.

## Figures and Tables

**Table 1:** pH variation in samples obtained from homemade yogurt on days 0, 7, 14, and 21 of storage.

Days	OYC	OY1	OY2	OY3	OY4
0	4.17 ^a^ ± 0.01	4.18 ^a^ ± 0.01	4.18 ^a^ ± 0.02	4.17 ^a^ ± 0.02	4.24 ^b^ ± 0.03
7	4.14 ^a^ ± 0.02	4.15 ^a^ ± 0.01	4.17 ^b^ ± 0.01	4.16 ^a,b^ ± 0.00	4.25 ^c^ ± 0.01
14	4.20 ^b^ ± 0.02	4.25 ^c^ ± 0.02	4.11 ^a^ ± 0.01	4.09 ^a^ ± 0.01	4.09 ^a^ ± 0.01
21	4.24 ^b^ ± 0.04	4.22 ^b^ ± 0.01	4.11 ^a^ ± 0.01	4.11 ^c^ ± 0.01	4.10 ^a^ ± 0.01

The data are presented as mean values (*n* = 3) ± standard deviation. Different superscript letters within the same parameter indicate significant differences between the experimental variants regarding their pH values (*p* < 0.05) at each time point. OYC = control yogurt; OY1 = yogurt with 0.1% potassium sorbate; OY2 = yogurt with 0.05% potassium sorbate and 0.05% EDB extract; OY3 = yogurt with 0.1% EDB extract; OY4 = yogurt with 0.2% EDB extract.

**Table 2:** Color characteristics of samples obtained from homemade yogurt, analyzed on day 0.

Sample	L*	a*	b*	C*	°hue
OYC	89.10 ^e^ ± 0.10	−2.48 ^a^ ± 0.02	8.72 ^e^ ± 0.05	9.06 ^b^ ± 0.05	105.85 ^d^ ± 0.08
OY1	88.47 ^d^ ± 0.18	−2.47 ^a^ ± 0.07	8.11 ^d^ ± 0.12	8.48 ^a^ ± 0.12	106.97 ^e^ ± 0.40
OY2	78.55 ^c^ ± 0.10	9.23 ^b^ ± 0.02	2.71 ^c^ ± 0.02	9.63 ^c^ ± 0.01	16.35 ^c^ ± 0.09
OY3	75.50 ^b^ ± 0.05	12.41 ^c^ ± 0.06	1.90 ^b^ ± 0.05	12.56 ^d^ ± 0.07	8.70 ^b^ ± 0.19
OY4	69.09 ^a^ ± 0.07	17.99 ^d^ ± 0.08	0.64 ^a^ ± 0.02	18.00 ^e^ ± 0.08	2.03 ^a^ ± 0.05

The data are presented as mean values (*n* = 3) ± standard deviation. Different superscript letters within the same parameter indicate significant differences between the experimental variants regarding their color characteristics (*p* < 0.05) at each time point. OYC = control yogurt; OY1 = yogurt with 0.1% potassium sorbate; OY2 = yogurt with 0.05% potassium sorbate and 0.05% EDB extract; OY3 = yogurt with 0.1% EDB extract; OY4 = yogurt with 0.2% EDB extract.

**Table 3:** Microbiological analysis of samples obtained from homemade yogurt: evolution of LAB, mesophilic bacteria, psychrophilic bacteria, yeasts, molds, and coliforms on 0, 7, 14, and 21 days of storage.

Day	Yogurt	LAB	Mesophilic Bacteria	Psychrophilic Bacteria	Yeasts and Molds	Coliform Bacteria
0	OYC	8.74 ^a,b^ ± 0.09	7.02 ^e^ ± 0.08	2.26 ± 0.06	1.72 ± 0.06	ND
	OY1	8.71 ^a^ ± 0.06	6.28 ^b^ ± 0.05	<1	<1	ND
	OY2	8.69 ^a^ ± 0.02	5.94 ^a^ ± 0.06	1.60 ± 0.03	<1	ND
	OY3	8.82 ^b^ ± 0.07	6.88 ^d^ ± 0.08	<1	<1	ND
	OY4	8.78 ^a,b^ ± 0.04	6.59 ^c^ ± 0.09	<1	<1	ND
7	OYC	8.33 ^a^ ± 0.10	7.18 ^b^ ± 0.05	2.30 ± 0.04	1.80 ± 0.02	ND
	OY1	8.32 ^a^ ± 0.06	6.32 ^a^ ± 0.09	<1	<1	ND
	OY2	8.31 ^a^ ± 0.05	6.35 ^a^ ± 0.04	<1	<1	ND
	OY3	8.29 ^a^ ± 0.08	7.59 ^d^ ± 0.07	<1	<1	ND
	OY4	8.25 ^a^ ± 0.05	7.32 ^c^ ± 0.02	<1	<1	ND
14	OYC	7.60 ^d^ ± 0.10	6.58 ^c^ ± 0.09	2.99 ± 0.07	2.04 ± 0.09	ND
	OY1	6.39 ^a^ ± 0.09	5.91 ^a^ ± 0.02	<1	<1	ND
	OY2	6.59 ^b^ ± 0.08	5.89 ^a^ ± 0.09	<1	<1	ND
	OY3	6.76 ^c^ ± 0.04	6.23 ^b^ ± 0.03	<1	<1	ND
	OY4	6.81 ^c^ ± 0.06	5.82 ^a^ ± 0.05	<1	<1	ND
21	OYC	6.90 ^d^ ± 0.09	5.43 ^d^ ± 0.07	3.71 ± 0.10	3.32 ± 0.07	ND
	OY1	6.37 ^b^ ± 0.03	4.62 ^b^ ± 0.04	<1	<1	ND
	OY2	5.34 ^a^ ± 0.04	5.67 ^e^ ± 0.05	<1	<1	ND
	OY3	6.49 ^c^ ± 0.07	5.23 ^c^ ± 0.07	<1	<1	ND
	OY4	6.51 ^c^ ± 0.04	4.34 ^a^ ± 0.04	<1	<1	ND

The data are presented as mean values (*n* = 3) of log CFU/g ± standard deviation. Different superscript letters within the same parameter indicate significant differences between the experimental variants (*p* < 0.05) at each time point. ND = not detected; OYC = control yogurt; OY1 = yogurt with 0.1% potassium sorbate; OY2 = yogurt with 0.05% potassium sorbate and 0.05% EDB extract; OY3 = yogurt with 0.1% EDB extract; OY4 = yogurt with 0.2% EDB extract.

**Table 4:** pH variation in samples obtained from commercial yogurt on days 0, 7, 14, and 21 of storage.

Days	CYC	CY1	CY2	CY3
0	4.51 ^a^ ± 0.02	4.53 ^a^ ± 0.02	4.50 ^a^ ± 0.01	4.53 ^a^ ± 0.01
7	4.55 ^a^ ± 0.02	4.57 ^b^ ± 0.01	4.58 ^b^ ± 0.01	4.54 ^a^ ± 0.01
14	4.61 ^b^ ± 0.02	4.61 ^b^ ± 0.02	4.62 ^b^ ± 0.01	4.59 ^a^ ± 0.01
21	4.57 ^c^ ± 0.01	4.54 ^b^ ± 0.01	4.56 ^c^ ± 0.00	4.53 ^a^ ± 0.01

The data are presented as mean values (*n* = 3) ± standard deviation. Different superscript letters within the same parameter indicate significant differences between the experimental variants regarding their pH values (*p* < 0.05) at each time point. CYC = control yogurt; CY1 = yogurt with 4% agave syrup; CY2 = yogurt with 0.5% EDB extract; CY3 = yogurt with 0.5% EDB extract and 4% agave syrup.

**Table 5:** Color characteristics of samples obtained from commercial yogurt, analyzed on day 0.

Sample	L*	a*	b*	C*	°hue
CYC	93.45 ^d^ ± 0.01	−1.75 ^a^ ± 0.02	10.04 ^c^ ± 0.03	10.19 ^a^ ± 0.03	99.87 ^b^ ± 0.08
CY1	92.88 ^c^ ± 0.01	−1.58 ^b^ ± 0.01	10.49 ^d^ ± 0.01	10.60 ^b^ ± 0.02	98.57 ^a^ ± 0.06
CY2	67.39 ^b^ ± 0.03	18.85 ^c^ ± 0.03	−2.40 ^a^ ± 0.02	19.00 ^c^ ± 0.03	352.73 ^c^ ± 0.07
CY3	66.45 ^a^ ± 0.03	19.05 ^d^ ± 0.03	−2.24 ^b^ ± 0.05	19.18 ^d^ ± 0.03	353.30 ^d^ ± 0.15

The data are presented as mean values (*n* = 3) ± standard deviation. Different superscript letters within the same parameter indicate significant differences between the experimental variants regarding their color characteristics (*p* < 0.05) at each time point. CYC = control yogurt; CY1 = yogurt with 4% agave syrup; CY2 = yogurt with 0.5% EDB extract; CY3 = yogurt with 0.5% EDB extract and 4% agave syrup.

**Table 6:** Microbiological analysis of samples obtained from commercial yogurt: evolution of LAB, mesophilic bacteria, psychrophilic bacteria, yeasts, molds, and coliforms on 0, 7, 14, and 21 days of storage.

Day	Yogurt	LAB	Mesophilic Bacteria	Psychrophilic Bacteria	Yeasts and Molds	Coliform Bacteria
0	CYC	6.46 ^b^ ± 0.04	6.31 ^a^ ± 0.04	<1	<1	ND
	CY1	6.34 ^a^ ± 0.08	6.31 ^a^ ± 0.04	<1	<1	ND
	CY2	6.30 ^a^ ± 0.07	6.30 ^a^ ± 0.06	<1	<1	ND
	CY3	6.40 ^a,b^ ± 0.03	6.29 ^a^ ± 0.02	<1	<1	ND
7	CYC	6.17 ^a^ ± 0.11	6.33 ^a^ ± 0.16	<1	<1	ND
	CY1	6.30 ^a^ ± 0.12	6.32 ^a^ ± 0.08	<1	<1	ND
	CY2	6.34 ^a^ ± 0.06	6.27 ^a^ ± 0.10	<1	<1	ND
	CY3	6.33 ^a^ ± 0.03	6.33 ^a^ ± 0.04	<1	<1	ND
14	CYC	6.25 ^a^ ± 0.02	6.25 ^a^ ± 0.04	<1	<1	ND
	CY1	6.28 ^a^ ± 0.08	6.24 ^a^ ± 0.03	<1	<1	ND
	CY2	6.22 ^a^ ± 0.06	6.31 ^a^ ± 0.06	<1	<1	ND
	CY3	6.18 ^a^ ± 0.03	6.26 ^a^ ± 0.03	<1	<1	ND
21	CYC	5.95 ^a^ ± 0.10	6.19 ^a^ ± 0.02	<1	<1	ND
	CY1	6.01 ^a^ ± 0.09	6.17 ^a^ ± 0.06	<1	<1	ND
	CY2	6.00 ^a^ ± 0.11	6.24 ^a^ ± 0.07	<1	<1	ND
	CY3	6.01 ^a^ ± 0.10	6.20 ^a^ ± 0.05	<1	<1	ND

The data are presented as mean values (*n* = 3) of log CFU/g ± standard deviation. Different superscript letters within the same parameter indicate significant differences between the experimental variants (*p* < 0.05) at each time point. ND = not detected; CYC = control yogurt; CY1 = yogurt with 4% agave syrup; CY2 = yogurt with 0.5% EDB extract; CY3 = yogurt with 0.5% EDB extract and 4% agave syrup.

## Data Availability

The original contributions presented in this study are included in the article. Further inquiries can be directed to the corresponding author.
